# Uni-ventricular palliation vs. bi-ventricular repair: differential inflammatory response

**DOI:** 10.1186/s40348-022-00138-y

**Published:** 2022-03-20

**Authors:** Matthias Sigler, Hatem Rouatbi, Jaime Vazquez-Jimenez, Marie-Christine Seghaye

**Affiliations:** 1 Pediatric Cardiology, Intensive Care Medicine and Neonatology, Georg-August Universität, Robert-Koch-Str. 40, D-37075 Göttingen, Germany; 2 Department of Pediatrics and Pediatric Cardiology, University Hospital Liège, Liège, Belgium; 3 Department of Pediatric Cardiac Surgery, University Hospital Aachen, Aachen, Germany

**Keywords:** Inflammatory balance, Tumor necrosis factor-α, Interleukin-6, Interleukin-10, Interleukin-12, Procalcitonin, Pediatric cardiac surgery, Total cavo-pulmonary connection, Bi-ventricular repair

## Abstract

**Background:**

To examine whether uni-ventricular palliation (UVP) and bi-ventricular repair (BVR) result in a different pattern of systemic inflammatory response to pediatric cardiac surgery with extra-corporeal circulation (ECC).

**Methods:**

In 20 children (median age 39.5 months) undergoing either UVP (*n* = 12) or BVR (*n* = 8), plasma levels of the inflammatory cytokines TNF-α, IL-6, IL-10, and IL-12 and of procalcitonin (PCT), were measured before, during and after open cardiac surgery up to postoperative day (POD) 10.

**Results:**

Epidemiologic, operative- and outcome variables were similar in both groups but post-operative central venous pressure that was higher in UVP. In the whole cohort, the inflammatory response was characterized by an early important, significant and parallel increase of IL-6 and IL-10 that reached their peak values either at the end of ECC (IL-10) or 4 h postoperatively (IL-6), respectively and by a significant and parallel decrease of TNF-α and IL-12 levels after connection to ECC, followed by a bi-phasic significant increase with a first peak 4 h after ECC and a second at POD 10, respectively. Patients after UVP showed a shift of the cytokine balance with lower IL-6- (*p* = 0.01) after connection to ECC, lower early post-operative TNF-α - (*p* = 0.02) and IL-12- (*p* = 0.04) concentrations and lower TNF-α/IL-10-ratio (*p* = 0.03) as compared with patients with BVR. Levels of PCT were similar in both groups.

**Conclusions:**

UVP is associated with an anti-inflammatory shift of the inflammatory response to cardiac surgery that might be related to the particular hemodynamic situation of patients with UVP.

## Introduction

Cardiac surgery with extra-corporal circulation (ECC) is associated with a systemic inflammatory reaction that comprises the release of pro- and anti-inflammatory mediators [1]. The inflammatory balance is thought to condition postoperative morbidity and mortality [2] and is influenced by numerous patient dependent and independent variables such as genetic predisposition, pre-operative hypoxemia or heart failure, complexity of surgery that impacts the duration and the quality of the whole management of ECC in terms of flow pattern, blood- and patient temperature, duration of myocardial ischemia, and peri-operative medication [3].

Besides circulating immune competent blood cells, other cell types such as endothelial-, myocardial-, and hepatic cells synthetize inflammatory mediators upon stimulation [4–7] and contribute to the inflammatory response to cardiac surgery. Cytokine signaling involves not only the classical infectious- and inflammatory- but also mechanical stress pathways that share common transcription pathways such as that of nuclear factor kappa B (NFκB) and activating protein (AP)-1 [8].

Hence, hemodynamic load that initiates mechanical stress of endothelial- or myocardial cells stimulates up-regulation of inflammatory genes [5, 6, 9]. In addition, systemic and pulmonary endothelial cells sense various flow patterns and respond via the activation pathway of NFκB [10]. Therefore, the characteristics of myocardial overload and of pulmonary flow in a patient before or immediately after the operation are expected to influence the inflammatory response to cardiac surgery. With this respect, hemodynamics after total cavo-pulmonary connection created for UVP-palliation that is characterized by at least a non-pulsatile pulmonary flow and increased central venous pressure with systemic and particularly hepatic vein congestion has the potential to modulate the peri-operative innate immune response [11]. This latter is complex and comprises the interplay of members of families of pro- and anti-inflammatory cytokines. Classical representative of pro-inflammatory cytokines are TNF-α and IL-12 [12, 13]. IL-6 has early pro- and later anti-inflammatory properties and is the main inductor of the acute phase reaction [14] whereas IL-10 is the major monocyte deactivating anti-inflammatory cytokine that contributes to termination of inflammation [15]. Given the inhibitory effect of IL-10 on the synthesis of pro-inflammatory cytokines, the inverse ratio between its levels and those of TNF-α are commonly used to reflect the inflammatory balance in diverse clinical situations [16]

In this study, we aimed to test the hypothesis that children undergoing UVP would show a different inflammatory balance than patients undergoing BVR. For this purpose, we analyzed data of a historical cohort of patients (surgery 1995–2000). We are aware that nowadays UVP is performed without cardioplegic arrest. Nevertheless, this similar surgical approach as well as a relatively homogenous hemodynamic situation preoperatively enabled us to directly compare the inflammatory response in patients undergoing UVP or BVR.

## Methods

### Patients

After approval by our local Human Ethical Committee and informed consent of the parents, 20 children aged 15–176 months, median 39.5 months with complex cardiac malformations were included in this prospective study. Twelve patients underwent UVP, 8 BVR. Characteristics of the patients groups are summarized in Table 1.

**Table 1:** Epidemiological and operative variables in children after uni-ventricular palliation or bi-ventricular repair

Variables	UVP	BV	*p* value
*n*	12	8	
Male/female (*n*)	7/5	7/1	NS
Age at operation (months)	39.5 (88.2)	39.5 (62.5)	NS
SaO_2_ (%)	78 (5.2)	84 (18.3)	NS
Cardiac malformation	SV (*n* = 7) TA (*n* = 3) Other (*n* = 2)	TOF (*n* = 5) PA-VSD (*n* = 3)	
Previous palliation	AP shunt *n* = 4 PAB *n* = 6	AP shunt *n* = 7	
Duration of ECC (min)	89.5 (82.8)	87 (47.8)	NS
Duration of aortic clamping (min)	69.8 (61.5)	67 (25.5)	NS
Water balance during ECC (ml)	135 (370)	162.5 (481)	NS

Values are displayed as median (IQR)

*Abbreviations: UVP* uni-ventricular palliation, *BVR* bi-ventricular repair, *SV* single ventricle, *TA* tricuspid atresia, *TOF* tetralogy of Fallot, *PA-VSD* pulmonary atresia with ventricular septal defect, *AP* aorto-pulonary, *PAB* pulmonary banding, *ECC* extracorporeal circulation

### Operation and post-operative care

In all cases, conventional general anesthesia consisted of isoflurane and sufentanyl. Dexamethasone (1 mg/m^2^ body surface area) was given before sternotomy. Perioperative antibiotic prophylaxis was carried out with cefuroxime. After institution of moderate hypothermic low-flow cardio-pulmonary bypass (CPB), the aorta was cross-clamped and cardiac arrest was instituted by intra-aortal injection of 4 °C cold cardioplegic solution (Bretschneider, 30 ml/kg body weight), which was re-aspirated in the right atrium. At the end of the intra-cardiac operative procedure, the patient was weaned from CPB under progressive re-warming. Epicardiac pace-maker leads and pericardial- and mediastinal drains were placed before chest closure.

Arterial blood pressure and central venous pressure were continuously monitored via an arterial- and a central venous line, respectively.

Inotropic support consisted of dobutamine given to maintain a normal mean arterial blood pressure for age and volume therapy by injections of crystalloid solutions, if requested.

The patient was transported on the intensive care unit where the weaning from the artificial ventilation was begun as early as possible. The ratio between arterial partial pressure of oxygen (PaO_2_) and fraction of inspired oxygen (FiO_2_) was used to assess oxygenation. Diuresis was continuously monitored via a bladder catheter as was water balance.

Routinely performed laboratory investigations included at least the determination of blood gases, blood concentration of lactate, glycemia, complete blood count, serum creatinine, aspartate aminotransferase (AST), coagulation parameters, and were measured at least 4- and 24 h post-operatively

### Blood samples

Venous blood was collected in EDTA tubes before the operation, 10 min after beginning of ECC, after protamine administration, 4 h post-operatively (4 h po), 1-, 2-, 3-, and 10 days after the operation (POD -1, -2, -3, and -10).

The samples were immediately centrifuged for 3 min (3000 rpm) and the plasma was stored at − 80 °C until analysis.

### Cytokine determination

TNF-α, IL-6, IL-10, and IL-12 were measured by enzyme amplified sensitivity immunoassay (EASIA, BioSource®, Belgium) according to the manufacturer’s recommendation.

### Procalcitonin (PCT)

PCT was determined using a specific immunoluminometric assay (Lumitest PCT, Brahms Diagnostica GmbH, Berlin, Germany). The detection limit of the method is 0.1 ng/ml. Normal values for healthy adults are < 0.1 ng/ml.

### Statistical analysis

Results are expressed by median and interquartile range (IQR) assuming not normal distribution of the data. For intergroup comparison of independent clinical and of biologic variables at specific sample times, the nonparametric Mann-Whitney ***U*** was used and the Wilcoxon test for the comparison of dependent variables when required. The Spearman rank correlation coefficient was assessed for correlation of independent parameters. ***P*** values < 0.05 were considered significant. Alpha adjustment for multiple comparisons was performed according to Bonferroni-Holm.

## Results

### Clinical data

There was no inter-group difference in epidemiologic- and operation data (Table 1) and post-operative clinical data (Table 2) but central venous pressure (CVP) at the end of ECC and 4 h po, that was significantly higher in patients after UVP than in patients after BVR (*p* = 0.01 and *p* = 0.039, respectively).

**Table 2:** Post-operative outcome variables in children after uni-ventricular palliation or bi-ventricular repair

Variables	UVP	BVR	*p* value
**Mean arterial pressure (mmHg)**			
4 h postoperatively (po)	58.5 (13)	63.2 (14.3)	NS
24 h po	69.2 (22.3)	62.5 (6.8)	NS
**Central venous pressure (mmHg)**			
4 h po	12.5 (2.3)	8.9 (2)	0.039
24 h po	13.0 (3.3)	11.0 (2)	NS
**Oxygenation index**			
4 h po	239.5 (202)	265 (355)	NS
24 h po	278 (239)	230 (327.5)	NS
**Water balance (mL/kg)**			
4 h po	55 (41.5)	56 (80)	NS
24 h po	52 (40)	51.5 (33.7)	NS
**ASL (unit/L)**			
4 h po	64.5 (79.5)	82.5 (24)	NS
24 h po	64 (51)	87 (45)	NS

Values are displayed as median (IQR)

*Abbreviations*: *UVP* uni-ventricular palliation, *BVR* bi-ventricular repair, *ASL* aspartate aminotransferase

### Laboratory results

#### TNF-α

In all patients, plasma levels of TNF-α fall after connection to ECC (*P* = 0.01). TNF-α increased during ECC (*p* = 0.01) to reach preoperative values 4 h po. TNF-α levels then decreased up to the POD 1 (*p* = 0.031) and stayed stable up to the POD 3, increasing again up to POD 10 to reach preoperative values.

**Fig. 1:**
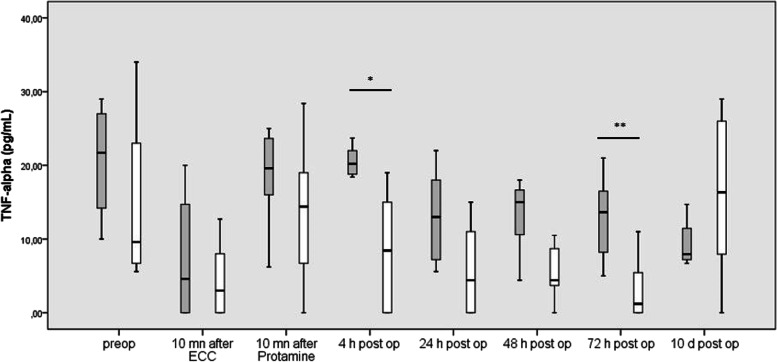
Blood concentrations of TNFα before, during and after cardiac surgery in patients undergoing uni-ventricular palliation (grey columns) and bi-ventricular repair (white column). Box plots indicate median with interquartile range

Patients after UVP showed significantly lower TNF-α values 4 h po and at POD 3 day than patients after BVR (*p* = 0.022 and *p* = 0.018, respectively), (Fig. 1).

TNF-α levels measured at the end of ECC correlated positively with water balance during ECC (*p* = 0.034) and negatively with the oxygenation index on the POD 1 (*p* = 0.06).

#### IL-12

**Fig. 2:**
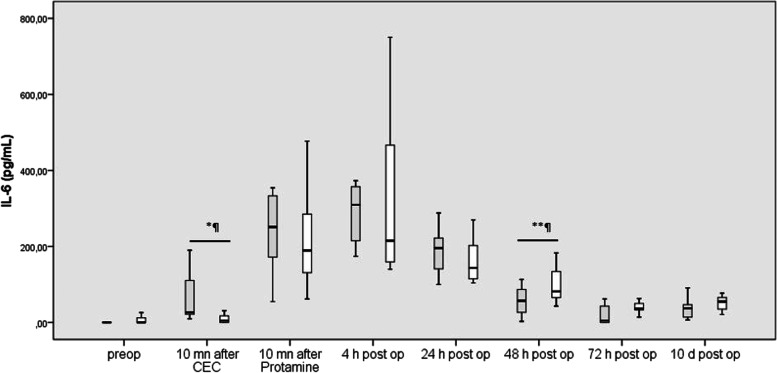
Blood concentrations of IL-6 before, during and after cardiac surgery in patients undergoing uni-ventricular palliation (grey columns) and bi-ventricular repair (white column). Box plots indicate median with interquartile range

In both groups, IL-12 levels decreased after connection to ECC (*p* = 0.0001) but increased during ECC (*p* = 0.0001) and further up to 4 h po (*p* = 0.046). IL-12 then decreased continuously in the first 3 post-operative days (*p* = 0.005, *p* = 0.06, and *p* = 0.006), respectively. Finally, IL-12 rose from POD 3 to POD 10 (*p* = 0.001), reaching near preoperative values (Fig. 2).

#### IL-6

In all patients, plasma levels of IL-6 rose after connection of ECC, increasing significantly during ECC (*p* = 0.001) and further to reach their peak value 4 h po (*p* = 0.031). IL-6 began to decrease continuously in the first 3 post-operative days (*p* = 0.008, *p* = 0.017, and *p* = 0.027 versus the previous value, respectively). IL-6 increased finally from POD 3 day until the POD 10 (*p* = 0.005).

**Fig. 3:**
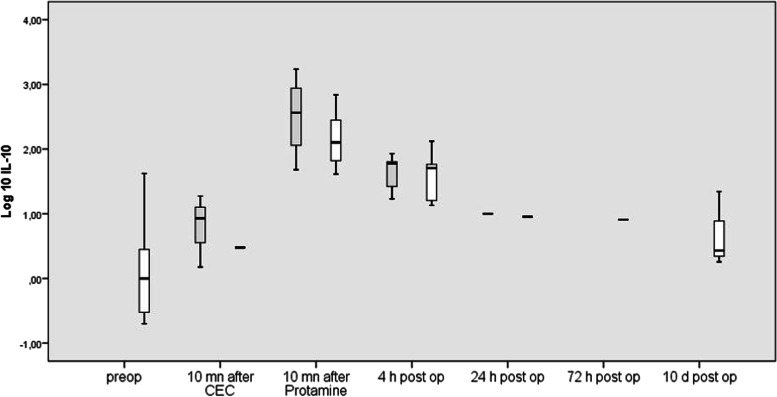
Blood concentrations of IL-10 before, during, and after cardiac surgery in patients undergoing uni-ventricular palliation (grey columns) and bi-ventricular repair (white column). Box plots indicate median with interquartile range. Results are shown as the decimal logarithm (missing logarithmic values resulted when absolute values were zero)

In patients after UVP, IL-6 levels were significantly lower immediately after connection of ECC and significantly higher at POD 2 than in patients after BVR (*p* = 0.011 and *p* = 0.048, respectively) (Fig. 3).

IL-6 levels measured after connection to ECC correlated negatively with CVP measured at the end of the operation (*p* = 0.001).

#### IL-10

In both groups, IL-10 levels rose significantly during ECC (*p* = 0.0001), reaching their peak value at the end of ECC. IL-10 levels then decreased abruptly in the first 4 h po (*p* = 0.0001) and further up to POD 1 (*p* = 0.0001) and POD 2 (*p* = 0.048). There was a secondary increase from POD 3 to POD 10 (*p* = 0.028).

**Fig. 4:**
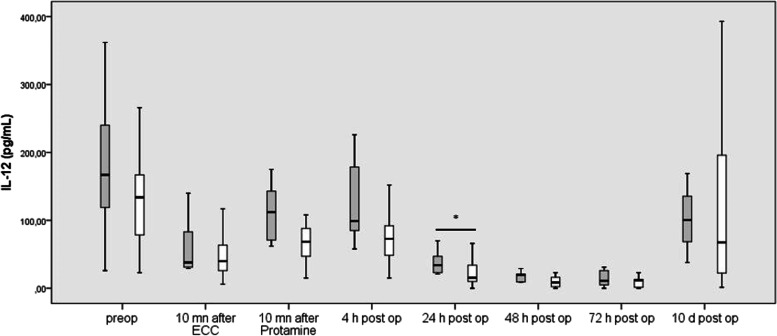
Blood concentrations of IL-12 before, during, and after cardiac surgery in patients undergoing uni-ventricular palliation (grey columns) and bi-ventricular repair (white column). Box plots indicate median with interquartile range

IL-10 blood levels were not different between both groups (Fig. 4).

IL-10 concentrations 4 h po correlated with TNF-α concentrations measured after ECC and 4 h po, respectively (*p* = 0.006 and *p* = 0.003, respectively).

IL-10 concentrations at the beginning of ECC correlated positively with mean arterial blood pressure 4 h po (*p* = 0.002). IL-10- and AST concentrations correlated negatively with each other 4 h po (*p* = 0.048).

#### PCT

PCT blood concentrations rose from the pre-operative period to 4 h po (*p* = 0.046), then further up to POD1 (*p* = 0.001), reaching their peak value and decreased slowly up to POD 10, where preoperative values were achieved. PCT levels were not different between groups.

PCT concentrations 4 h po correlated significantly with TNF-α levels at the end of ECC and 4 h po (*p* = 0.037 and *p* = 0.019, respectively), with IL-6 levels at the end of ECC and 4 h po (*p* = 0.009 and *p* = 0.008, respectively), with IL-10 levels at the end of ECC (*p* = 0.013).

PCT levels did not correlate with clinical outcome variables.

## Discussion

Our results confirm that cardiac surgery in children induces a systemic release of the pro- and anti-inflammatory cytokines TNF-α, IL-6, IL-10, and IL-12 that persists at least up to POD 5, and that the importance of the inflammatory response is well reflected by the early post-operative PCT levels.

TNF-α is an early pro-inflammatory cytokine that has also a role in the maintenance of homeostasis [12]. It induces among others IL-6 [17]. TNF-α and IL-6 are implicated in the pathophysiology of heart failure and of pulmonary hypertension [18]. IL-6 helps terminating inflammation via the induction of C-reactive protein in the liver and via the induction of the monocyte deactivating cytokine IL-10 [15]. The liver is an important source of IL-10 during cardiac surgery, as we showed previously [7]. IL-10 inhibits the synthesis of all pro-inflammatory cytokines via the activation of the suppressor of cytokine signaling (SOCS) [19]. SOCS in turn is modulated by mechanical stress as it has been shown in human endothelial cells [20].

IL-12 is a pro-inflammatory cytokine that is produced by antigen presenting cells such as dendritic cells and macrophages. It regulates interferon-γ (INFγ) production by Th1 lymphocytes [13]. In adults and children undergoing cardiac surgery, decreased ex vivo IL-12 production has been demonstrated [21, 22], thus favoring anti-inflammatory balance [23].

In the present series, upon connection with the ECC circuit, the concentrations of TNF-α and IL-12 fall significantly. This decrease may be the result of the combined effects of dilution and corticosteroids given before the operation [24]. Both pro-inflammatory cytokines showed a similar course with a secondary significant increase of their blood concentrations during ECC and a peak value measured 4 h po, reflecting the pro-inflammatory effect of cardiac surgery [3]. The correlations between TNF-α levels at the end of ECC and the volume of water retention during ECC and the oxygenation index on POD 1, respectively, confirm the harmful impact of inflammation on endothelial- and organ function [3]. The second decrease of TNF-α- and IL-12 concentrations with a nadir on POD 3 reflects the counteracting effect of the anti-inflammatory response [25]. This latter was objectivized in this series by an early and significant increase of IL-10 blood concentrations reaching their peak value at the end of ECC. Interestingly, the importance of the early release of IL-10 was associated with higher mean arterial blood pressure in the early post-operative period, reflecting the clinical relevance of the anti-inflammatory response during cardiac surgery.

IL-6 showed a similar course to IL-10. IL-6 has early pro- and later anti-inflammatory properties as well and its blood levels usually reflect the amount of systemic inflammation [26]. In this series, its early release after connection to ECC was associated with lower CVP, confirming the role of inflammation in the pathophysiology of capillary leakage during cardiac surgery [27].

The comparison of the time course of TNF-α and IL-12 blood concentrations on the one hand and of IL-6 and IL-10 on the other hand is suggestive for the interplay between pro- and anti-inflammatory signals during and after cardiac surgery [1]. This is supported by the significant correlations between TNF-α and IL-10 concentrations we observed in the early post-operative period.

The TNF-α/IL-10 ratio can be considered a marker of the inflammatory balance [28]. In this series, higher ratio correlated with lower water balance during CPB, confirming again the role of inflammation in capillary leakage during cardiac surgery.

The main objective of this study was to test the hypothesis that patients with UVP circulation would respond differently to cardiac surgery in terms of systemic inflammation than patients undergoing BVR. Our results allow suggesting such a differential response. Indeed, patients after UVP showed significantly lower concentrations of TNF-α and IL-12 with higher IL-6 blood levels and lower TNF-α/IL-10-ratio in the early post-operative period than BVR patients. This is indicative for a shift of the inflammatory balance towards anti-inflammation in patients with UVP.

The systemic inflammatory reaction elicited by cardiac surgery is complex and influenced by numerous patient-dependent or patient-independent variables [3]. With this respect, intra- and post-operative hemodynamics is potentially an important influencing factor [29]. Patients with UVP have as main hemodynamic characteristics high CVP with distension of the systemic venous system and non-pulsatile arterial pulmonary blood flow. Our results showing higher CVP in the early po period in UVP than BVR patients are concordant with this.

Mechanical stretch encountered in arterial and venous vessels under normal or pathological conditions have a direct impact on endothelial cell function throughout the activation of several transcription pathways such as that of NFκB and AP-1 that are common to the inflammation pathways [30, 31].

Thus, high pulsatility flow due to vascular stiffening has been shown to induce significant acute and sustained endothelial inflammation mediated by the activation of NFκB whereas low pulsatility flow was associated with only minor and transient inflammation [32]. Not only flow pulsatility but also the helical flow structure initiates inflammatory signals in endothelial cells via NFκB activation, leading to the downstream synthesis of IL-1β, TNF-α, IL-6, and INFγ [9].

Helical flow structure has been demonstrated in the right artery after cavo-pulmonary connection [33] but whether it influences local or the systemic inflammatory pathways has not been investigated yet.

Besides the impact of flow pattern on the endothelial inflammatory signals, mechanical stretch of myocardial cells due to hemodynamic overload of cardiac cavities induces an intra-myocardial expression of pro- and anti-inflammatory cytokines, as we showed previously in children with congenital cardiac defect [5]. These inflammatory mediators are released into the circulation [34] and may participate to the inflammatory response to cardiac surgery and influence post-operative outcome.

### Limitations

The small patient number that did not allow to test the possible relationship between hemodynamics and markers of inflammation during and after the operation is the main limitation of our study.

It is of note that surgery for the patients described in our study was performed in the years 1995 to 2000. Surgical techniques have been substantially modified and evolved in the meantime. But it was the relatively homogenous group of patients (with pulsatile flow characteristics in the pulmonary circulation preoperatively in all patients) that enabled us to compare the differential cytokine response between patient undergoing UVP or BVR.

## Conclusion

Patients undergoing UVP shift their inflammatory response to cardiac surgery towards anti-inflammation. This might be due to particular intra-cardiac and pulmonary flow pattern and hemodynamics characteristic for this patient population.

## Data Availability

All data generated or analyzed during this study are included in this published article.

## Abbreviations

AP: Activating protein
AST: Aspartate aminotransferase
BVR: Bi-ventricular repair
CPB: Cardio-pulmonary bypass
CVP: Central venous pressure
ECC: Extra-corporeal circulation
FiO_2_: Fraction of inspired oxygen
INFγ: Interferon-γ
NFκB: Nuclear factor kappa B
PaO_2_: Arterial partial pressure of oxygen
PCT: Procalcitonin
POD: Postoperative day
SOCS: Suppressor of cytokine signaling
UVP: Uni-ventricular palliation

## References

[1] Hövels-Gürich H, Schumacher K, Vazquez-Jimenez J (2002). Cytokine balance in infants undergoing cardiac operation. AnnThorac Surg.

[2] Hövels-Gürich H, Vazquez-Jimenez J, Silvestri A (2002). Production of proinflammatory cytokines and myocardial dysfunction after arterial switch operation in neonates with transposition of the great arteries. J Thorac Cardiovasc Surg.

[3] Seghaye M (2003). The clinical implications of the systemic inflammatory reaction related to cardiac operations in children. Cardiol Young.

[4] Buchko M, Stewart C, Hatami S, Himmat S, Freed D, Nagendran J (2019). Total parenteral nutrition in ex vivo lung perfusion: addressing metabolism improves both inflammation and oxygenation. American Journal of Transplantation.

[5] Rouatbi H, Farhat N, Heying R, Gérard A, Vazquez-Jimenez J, Seghaye M (2020). Right atrial myocardial remodeling in children with atrial septal defect involves inflammation, growth, fibrosis, and apoptosis. Front Pediatr.

[6] Qing M, Schumacher K, Heise R (2003). Intramyocardial synthesis of pro- and anti-inflammatory cytokines in infants with congenital cardiac defects. J Am Coll Cardiol.

[7] Qing M, Nimmesgern A, Heinrich P (2003). Intrahepatic synthesis of tumor necrosis factor-α related to cardiac surgery is inhibited by interleukin-10 via the Janus kinase (Jak)/signal transducers and activator of transcription (STAT) pathway. Crit Care Med.

[8] Glossop J, Cartmell S (2009). Effect of fluid flow-induced shear stress on human mesenchymal stem cells: Differential gene expression of IL1B and MAP 3K8 in MAPK signaling. Gene Expression Patterns.

[9] Liu S, Deng X, Zhang P (2020). Blood flow patterns regulate PCSK9 secretion via MyD88-mediated pro-inflammatory cytokines. Cardiovasc Res.

[10] Nakajima H, Mochizuki N (2017). Flow pattern-dependent endothelial cell responses through transcriptional regulation. Cell Cycle.

[11] Lommi J, Pulkki K, Koskinen P (1997). Haemodynamic, neuroendocrine and metabolic correlates of circulating cytokine concentrations in congestive heart failure. Eur Heart J.

[12] Sedger L, McDermott M (2014). TNF and TNF-receptors: From mediators of cell death and inflammation to therapeutic giants – past, present and future. Cytokine Growth Factor Rev.

[13] Sun L, He C, Nair L, Yeung J, Egwuagu C (2015). Interleukin 12 (IL-12) family cytokines: Role in immune pathogenesis and treatment of CNS autoimmune disease. Cytokine.

[14] Tanaka T, Narazaki M, Kishimoto T (2014). IL-6 in inflammation, immunity, and disease. Cold Spring Harb Perspect Biol.

[15] Walter M (2014). The molecular basis of IL-10 function: from receptor structure to the onset of signaling. Curr Top Microbiol Immunol.

[16] Goswami B, Rajappa M, Mallika V, Shukla D, Kumar S (2009). TNF-α/IL-10 ratio and C-reactive protein as markers of the inflammatory response in CAD-prone North Indian patients with acute myocardial infarction. Clinica Chimica Acta.

[17] Luo Y, Zheng SG (2016). Hall of fame among pro-inflammatory cytokines: Interleukin-6 gene and its transcriptional regulation mechanisms. Front Immunol.

[18] Groth A, Vrugt B, Brock M, Speich R, Ulrich S, Huber LC (2014). Inflammatory cytokines in pulmonary hypertension. Respir Res.

[19] Murray PJ (2007). The JAK-STAT signaling pathway: input and output integration. J Immunol.

[20] Dangers M, Kiyan J, Grote K, Schieffer B, Haller H, Dumler I (2010). Mechanical stress modulates SOCS-1 expression in human vascular smooth muscle cells. J Vasc Res.

[21] Franke A, Lante W, Kurig E, Zöller LG, Weinhold C, Markewitz A (2006). Is interferon gamma suppression after cardiac surgery caused by a decreased interleukin-12 synthesis?. Ann Thorac Surg.

[22] Justus G, Walker C, Rosenthal L-M, Berger F, Miera O, Schmitt KRL (2019). Immunodepression after CPB: cytokine dynamics and clinics after pediatric cardiac surgery - a prospective trial. Cytokine.

[23] De Angelis E, Pecoraro M, Rusciano MR, Ciccarelli M, Popolo A (2019). Cross-talk between neurohormonal pathways and the immune system in heart failure: a review of the literature. Int J Mol Sci.

[24] Bronicki RA, Backer CL, Baden HP, Mavroudis C, Crawford SE, Green TP (2000). Dexamethasone reduces the inflammatory response to cardiopulmonary bypass in children. Ann Thorac Surg.

[25] Heying R, Wehage E, Schumacher K (2012). Dexamethasone pretreatment provides antiinflammatory and myocardial protection in neonatal arterial switch operation. Ann Thorac Surg.

[26] Brocca A, Virzì GM, de Cal M, Giavarina D, Carta M, Ronco C (2017). Elevated levels of procalcitonin and interleukin-6 are linked with postoperative complications in cardiac surgery. Scand J Surg.

[27] Seghaye MC, Grabitz RG, Duchateau J (1996). Inflammatory reaction and capillary leak syndrome related to cardiopulmonary bypass in neonates undergoing cardiac operations. J Thorac Cardiovasc Surg.

[28] Tsurumi A, Que Y-A, Ryan CM, Tompkins RG, Rahme LG (2016). TNF-α/IL-10 ratio correlates with burn severity and may serve as a risk predictor of increased susceptibility to infections. Front Public Health.

[29] Zorzanelli L, Maeda NY, Clavé MM, Aiello VD, Rabinovitch M, Lopes AA (2016). Serum cytokines in young pediatric patients with congenital cardiac shunts and altered pulmonary hemodynamics. Mediators Inflamm.

[30] Chiu J-J, Chien S (2011). Effects of disturbed flow on vascular endothelium: pathophysiological basis and clinical perspectives. Physiol Rev.

[31] Demicheva E, Hecker M, Korff T (2008). Stretch-induced activation of the transcription factor activator protein-1 controls monocyte chemoattractant protein-1 expression during arteriogenesis. Circ Res.

[32] Li M, Tan Y, Stenmark KR, Tan W (2013). High pulsatility flow induces acute endothelial inflammation through overpolarizing cells to activate NF-κB. Cardiovasc Eng Technol.

[33] Sundareswaran KS, Haggerty CM, de Zélicourt D (2012). Visualization of flow structures in Fontan patients using 3-dimensional phase contrast magnetic resonance imaging. J Thorac Cardiovasc Surg.

[34] Bartekova M, Radosinska J, Jelemensky M, Dhalla NS (2018). Role of cytokines and inflammation in heart function during health and disease. Heart Fail Rev.

